# Effects of *miR-338* on morphine tolerance by targeting CXCR4 in a rat model of bone cancer pain

**DOI:** 10.1042/BSR20160517

**Published:** 2017-03-15

**Authors:** Hong-Xia Mei, Min-Hong Zhou, Xing-Wang Zhang, Xi-Xi Huang, Yong-Le Wang, Pei-Fang Wang, Gong-Hao Zhan

**Affiliations:** 1 Department of Anesthesiology, The Second Affiliated Hospital and Yuying Children’s Hospital of Wenzhou Medical University, Wenzhou 325027, P.R. China; 2 Department of Pain Management, The Second Affiliated Hospital and Yuying Children’s Hospital of Wenzhou Medical University, Wenzhou 325027, P.R. China; 3 Department of Pharmaceutics, The Pharmacy College of Jinan University, Guangzhou 510632, P.R. China; 4 Department of Orthopedics, Wenzhou Province Hospital of TCM, Wenzhou 325027, P.R. China

**Keywords:** bone cancer pain, CXCR4, MiR-338, morphine tolerance, PLV-THM-miR-338 lentivirus, PNL-RiCXCR4 lentivirus

## Abstract

The present study aimed to investigate the effects of *miR-338* on morphine tolerance through the targeting of CXC chemokine receptor-4 (CXCR4) in a rat model of bone cancer pain (BCP). Sprague–Dawley (SD) rats were obtained and divided into model saline (*n*=10), model morphine (*n*=50), normal saline (*n*=10) and normal morphine (healthy rats, *n*=10) groups. After BCP rat model establishment, the remaining SD rats (*n*=40) in the model saline group were assigned into pLV-THM-*miR-338*, pLV-THM-anti-*miR-338*, CXCR4 shRNA, blank and PBS groups. Luciferase reporter gene assay was used for luciferase activity. Quantitative real-time PCR (qRT-PCR) and Western blotting were performed to detect the *miR-338* and CXCR4 mRNA and protein expression. The model saline group showed increased mRNA and protein expressions of CXCR4 but decreased *miR-338* compared with the model saline group, and the model morphine group had increased mRNA and protein expressions of CXCR4 but decreased *miR-338* compared with the model saline group. The mRNA and protein expressions of *miR-338* in the pLV-THM-*miR-338* group increased remarkably while those of the pLV-THM-anti-*miR-338* group decreased significantly compared with the CXCR4 shRNA, blank and PBS groups. The pLV-THM-*miR-338*, pLV-THM-anti-*miR-338*, CXCR4 shRNA and CXCR4 mRNA groups all had lower mRNA and protein expressions of CXCR4 than those in the blank and PBS groups. *miR-338* exerts significant influence in the inhibition of morphine tolerance by suppressing CXCR4 in BCP.

## Introduction

Bone cancer pain (BCP) is quite complex and a recent systematic review has found that the incidence of advanced or metastatic disease in patients with cancer pain is 64% [1]. Bone metastases in advanced cancer frequently cause painful complications, and for 36–50% of cancer patients this pain is severe enough to compromise their daily lives [2]. The most common causes of BCP are breast cancer and prostate cancer, with bone absorption and cancers being the primary factors for pain [3]. Clinically, morphine and other opioids are typically used in BCP treatment; however, prolonged morphine treatment leads to morphine tolerance, resulting in a reduction in pain-suppression effects, shorter effective time and increased pain sensitivity in patients, while the specific mechanisms behind morphine tolerance are not yet clear [4]. CXC chemokine receptor-4 (CXCR4) signalling has been shown to contribute to the maintenance and development of BCP by activating astrocytes and microglia as well as sensitizing neurons [5], and *miR-622* and *miR-146a* were found to be related to CXCR4 expression [6,7].

miRNA is a type of small non-coding RNA molecule, modulating gene expression by targeting mRNA and triggering decoding inhibition or RNA degradation [8]. As a brain-specific miRNA, *miR-338* is located in the eighth intron of apoptosis-associated tyrosine kinase (AATK) [9], and it is believed to target pathways in cells proliferation and differentiation [10]. One study showed that *miR-338* overexpression in cancer cells is abnormal [11], and reduces cell metastasis, invasion, proliferation and apoptosis [12].* miR-338-3p*, as a subgroup of *miR-338*, is also understood to inhabit cancer genes in various cancers [13]. Chemokine receptor is a widely expressed G-protein-coupled receptor, and is related to a number of human diseases such as HIV and cancers, in a process related to CVCR4 signalling pathway’s disturbance [14]. Chemokine receptor signals become specific CXCR4 receptor antagonists through the CXCR4 receptor, which may play a significant role in opioid-induced pain and could affect the prior role of morphine treatment for severe pain [15]. There have been reports on miRNAs and their regulation of CXCR4 [16], yet literature on *miR-338*’s targeting of CXCR4 is not widely available. Thus, this research created a BCP rat model to assess the mechanisms of *miR-338* targeting CXCR4 during the formation of morphine tolerance in BCP.

## Materials and methods

### Ethical statement

This experiment was performed strictly in accordance with the research outlines on the use of awake animals in pain studies of the International Association for the Study of Pain (IASP). The experimental methods were approved by the Ethics Committee on animal testing of The Second Affiliated Hospital and Yuying Children’s Hospital of Wenzhou Medical University.

### Establishment of BCP models

Sixty clean and healthy female adult Sprague–Dawley (SD) rats weighing 180–210 g in clean state were obtained from Guangdong Provincial Experimental Animal Center (Animal licence No. SCXK 2008-0002, Guangdong, China). The rats were intraperitoneally injected with (5 ml/kg) 896 chloral hydrate for anaesthesia. After this, the right knee joints were shaved and the skin was disinfected with 70% alcohol. Knee joints were fixed using the left hand so that the surface skin was stretched. Then, needle 7 was used to drill at the knee joints (the edge of skeletal ligament) along the tibial longitudinal axis to the distal end of tibia for a distance of 1 cm. Needle 5 with microinjector was used to inject tumour cells (4 × 10^5^) to the tibial bone marrow cavity, after which intraperitoneal injections of gentamicin of recommended dosage were given for the following consecutive 3 days to prevent infection.

### Establishment of BCP rat models with morphine tolerance

Sixty rats of BCP models were randomly divided into model saline (physiological saline injection, *n*=10) and model morphine (morphine injection, *n*=50) groups. Another 20 normal rats were randomly divided into normal saline (healthy rats, *n*=10) and normal morphine (healthy rats, *n*=10) groups. Subcutaneous injection of 10 mg/kg morphine was performed on rats in the model morphine and normal morphine groups [17]. The injection was given twice a day, at 8:00 and 18:00, for seven consecutive days. Subcutaneous injection of normal saline with equal volume was also performed based on rat body weight. After the injection, ten rats in each group were killed on the 7th day to detect expressions of *miR-338* and CXCR4. The remaining 40 rats in the model morphine group were used for further experimentation.

### Plasmid construction

Three plasmids were adopted for the expression system, namely transfer plasmid pGLV-H1-GFP + Puro, coated plasmid PG-P1-VSVO and packaging plasmid PG-P2-REV. Recombinant pLV-THM-*miR-338* and CXCR4 shRNA lentiviral vector were synthesized by Shanghai GenePharma Co., Ltd. (Shanghai, China) and verified by sequencing. The sequence of pLV-THM-*miR-338* was 5′-CAACAAAAUCACUGAUGCUGGA-3′ and the plasmid sequence of CXCR4 shRNA was 5′-AGACTGATGAAGGCCAGGATT-3′. HEK293T cells (0.5 × 10^4^/well) were inoculated in each well within a 96-well plate, and cultured overnight in 100 μl of Dulbecco’s modified Eagle’s medium (DMEM) containing 10% FBS. DMEM with 10% FBS and 5 μg/ml polybrene was used to dilute lentivirus in every group into different gradients on the following day. After removing the cell culture, 100 μl of virus diluent was added to each well, and three replicated wells were set for each dilution factor. The cells were cultured overnight. The following day, 100 μl of complete medium was added for 48-h culturing, after which the culture was removed. Finally a microscope was used to count GFP light emitting positive transduction unit (TU), and virus titre was calculated using the formula TU/ml = [(infected cells/field) × (fields/well)]/volume virus (ml) × dilution factor.

### Grouping of BCP rat models with morphine tolerance and lentivirus infection

The remaining 40 rat models were randomly divided into the pLV-THM-miR-338, CXCR4 shRNA, blank control and PBS control groups. Intravenous injection of virus suspension of the same titre or equivalent sterile PBS solution into the tail was performed on rats in every group. After successful establishment of BCP rat model with morphine tolerance, specifically the 7th day after morphine injection, 50% mechanical withdrawal threshold (MWT) was measured. Finally, 50% MWT of each group was measured on the 7th, 9th and 14th day after injection.

### Luciferase reporter gene assay

The mutant and wild-type sequence of 3′-UTR of *CXCR4* gene was connected to the dual luciferase reporter gene vector to construct the recombinant plasmids pmiR-PB-Report^TM^ Vector-CXCR4-3′-UTR and pmiR-PB-Report^TM^ Vector-CXCR4-3′-UTR-Del, which were then divided into pmiR-PB-Report^TM^ Vector-CXCR4-3′-UTR, *miR-338* negative control (NC), pmiR-PB-Report^TM^ Vector-CXCR4-3′-UTR, miR-338mimics, pmiR-PB-Report^TM^ Vector-CXCR4-3′-UTR, miR338 inhibitor, pmiR-PB-Report^TM^ Vector -CXCR4-3′-UTR-Del, miR338 mimics, pmiR-PB-Report^TM^ Vector-CXCR4-3′-UTR-Del, miR-338 inhibitor, pmiR-PB-Report Vector -CXCR4-3′-UTR-Del and miR338 NC groups. In a 96-well plate, a total of 1.5 × 10^4^ cells were inoculated in every well with 100 μl of medium and cultured in the incubator with 5% CO_2_ and saturated humidity at 37°C for 24 h. The next day, the medium in every well was replaced with 50 μl of medium, after which 10 μl of anti-serum medium OPTI-MEM (Thermo Fisher Scientific Inc., Waltham, MA, U.S.A.) was used to dilute *miR-338* mimics to 100 nmol/l, 15 μl of OPTI-MEM to dilute recombinant plasmid CXCR4-WT or CXCR4-Mut to 100 ng and 25 μl of OPTI-MEM to dilute Lipofectamine 2000 to 0.25 μl. After 5 min, the diluted solution was mixed, gently shaken and placed at room temperature for 20 min. Then, 50 μl of the mixed liquor was added into the well until the total volume of every well reached 100 μl. Three replicated wells were set in every group. After being transfected for 6 h, 100 μl of fresh medium was added. After 48-h transfection, luciferase reporter gene assay kit (Shanghai Beyotime Biotechnology Co. Ltd., Shanghai, China) was used for dual luciferase reporter gene assay and relative luciferase activity (hRluc/hLuc) was compared among the different groups.

### Behavioural test

The BCP rat model was established according to the methods of a previous study [18]. MWT was used for assessment of behaviour. The rats were placed in a 26 cm × 14 cm × 26 cm transparent glass box, with wire net frame (0.5 cm × 0.5 cm × 22 cm). After placing the rats inside and allowing them to exercise freely or rest for 15–20 min, standardized von Frey cilia (Stoelting, Philadelphia, PA, U.S.A.) (at a density of 0.18, 0.25, 0.6, 1.3, 3.8, 5.4, 7.6 and 9.7 g) was respectively used to stimulate the postmedian of the rat foot vertically for 6–8 s. All intensities above were repeated five times and the second stimulation was performed 2 min after the first, once its reaction had totally disappeared. It can be drawn from this that rat withdrawal threshold to mechanical stimulation (50% MWT) = the minimum von Frey fibre strength among more than two paw withdrawal.

### Specimen collection and fluorescence microscopy

Rats in each group were anaesthetized with pentobarbitone sodium and underwent thoracotomy, after which cannulae were used in the CV ascending aorta, delivering 20 ml of 0.9% saline to wash the blood quickly and 20 ml of 0.1 mol/l PBS (pH = 7.4) with 4% paraformaldehyde for perfusion fixation for 20 min. After perfusion, L5 spinal cord was placed in fresh fixative at 4°C for 4–6 h and transferred to 20% and 30% sucrose solution for soaking until it sank to the bottom. PBS was used to substitute the primary antibody in the NC. After rinsing with PBS, goat-anti-rabbit IgG-labelled with FITC was used as the corresponding secondary antibody, being incubated at room temperature in darkness for 2 h. The slices were placed under OLYMPUS IX81 light microscope (Olympus, Tokyo, Japan) and stained with a fluorescent dye Hoechst 33258 (Sigma–Aldrich Chemical Company, St. Louis, MO, U.S.A.). The coverslip was placed with cells facing down and was mounted with 50% glycerol, put on its slide, then observed and photographed under the oil immersion lens of fluorescence microscope (Leica DMIRB).

### Quantitative real-time PCR

L_3-4_ segment tissues of rats, following successful construction of BCP rat model with morphine tolerance and lentiviral transfection, were dealt and mixed with liquid nitrogen into powder. Total RNA was extracted from the tissues with Trizol (Gibco Company, Grand Island, NY, U.S.A.) according to the instructions. The concentration and purity of RNA were detected with UV spectrophotometer. The extracted 1000 ng of RNA samples were used to construct 20 μl of reverse transcription system with 4 μl of 5× PrimeScript^TM^ Buffer, 1 μl of PrimeScript^TM^ RT Enzyme Mix I, 1 μl of OligodT Primer (50 uM), 1 μl of Random 6 mers (100 µM) and RNase Free dH_2_O according to the instructions of PrimeScript^TM^ RT Regeant Kit (Takara Biotechnology Ltd., Liaoning, China). Reverse transcription was conducted at 37°C for 30 min and then 85°C for 6 s. SYBR Premix Ex Taq II kit (Takara Biotechnology Ltd., Liaoning, China) was used for quantitative real-time PCR (qRT-PCR). One microlitre of reverse transcription product was used to construct 20 μl of reaction system with 10 μl of SYBR Green I Premix Ex Taq II (2×), 0.8 μl of PCR Forward Primer (10 µM), 0.8 μl of PCR Reverse Primer (10 µM), 0.4 μl of ROX Reference Dye (50×) and 7 μl of dH_2_O. The reverse transcription proceeded with initial denaturation at 95°C for 30 s, then 40 cycles with denaturation at 95°C for 5 s, annealing at 56°C for 30 s and extension at 72°C for 30 s. Finally, melt expression was performed. Relative expression of the target gene was calculated using 2^−ΔΔ*C*^_T_ method. The experiment was repeated three times on every sample. The internal reference genes of *miR-93*-5p and Smad5 were U6snRNA and glyceraldehyde-3-phosphate dehydrogenase (GAPDH) respectively. The primer sequences are shown in Table 1.

**Table 1:** The primer sequences of qRT-PCR

Gene		Sequence
*mir-338*	F:	5′-AACAAUAUCCUGGUGCUGAGUG-3′
	R:	5′-CUCAGCACCAGGAUAUUGUUUU-3′
U6snRNA	F:	5′-ATTGGAACGATACAGAGAAGATT-3′
	R:	5′-GGAACGCTTCACGAATTTG-3′
CXCR4	F:	5′-CTTACTACATTGGGATCAGC-3′
	R:	5′-AGTCCTACCACGAGACATAC-3′
GAPDH	F:	5′-TCATGGGTGTGAACCATGAGAA-3′
	R:	5′-GGCATGGACTGTGGTCATGAG-3′

Note: F, forward; R, reverse.

### Western blotting

After the hearts of rats were perfused with 20 ml of normal saline, L_5_ segment of the spinal cord was removed and placed in a 2 ml centrifuge tube treated with diethyl pyrocarbonate (DEPC) solution. After this 100 μl of radio-immunoprecipitation assay (RIPA) cell lysis buffer (ShineGene Molecular Biotechnology, Shanghai, China) was added to the electric homogenate. The samples were then put on ice statically for 30 min after which the total cell proteins were extracted according to the instructions. BCA protein assay kit (Beyotime Biotechnology Co., Shanghai, China) was used to quantify the extracted protein. SDS/PAGE board was prepared and the sample volume was calculated on the basis of the protein concentration. The sample was mixed with sample buffer (1:1) and put into boiling water for 5 min. Under conditions of 80 and 100 V, 5% concentrated gel and 12% separate gel were used for electrophoresis. The gelatin was cut, marked by cutting the corners off to make a ‘sandwich’ structure and arranged for electroporation in refrigerator at 4°C with a constant current of 200 mA for 2 h. The samples were sealed for 1 h at room temperature with the addition of 5% skimmed milk powder, after which the membrane was washed with TBS tween (TBST) for 10 min with three repetitions and immersed in 1:1000 rabbit anti-CXCR4 monoclonal antibody (ab92698 and Abcam, U.S.A.) and 1:4000 rabbit-anti-human β-actin polyclonal antibody (ab129348, Abcam Inc., Cambridge, MA, U.S.A.) respectively for incubation at 4°C overnight. The following day, the membrane was washed for 10 min with three repetitions and put in horseradish peroxidase (HRP)-marked sheep-anti-rabbit IgG (Sigma–Aldrich Chemical Company, St. Louis, MO, U.S.A.) for incubation at room temperature for 1 h. The membrane was then developed with ECL kit and scanned with gel imaging system. The bands obtained were analysed with ImageJ software. The relative expression of CXCR4 in each group was compared, with β-actin serving as internal control.

### Statistical analysis

Statistical analyses were conducted using SPSS20.0 software. All measurement data were expressed by means and standard deviations. One-way ANOVA was used to compare behaviour indices and mRNA expressions of *miR-338* and CXCR4 of rats at specific time points among different groups. Variance analysis of repeated measurement data was used for comparison within one group. *P*<0.05 was considered as statistically significant.

## Results

### Comparisons of MWT detected by behavioural test among four groups

**Figure 1:**
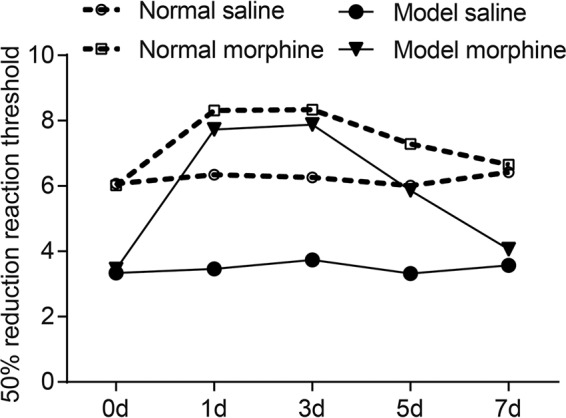
Comparisons of MWT detected by behavioural test among four groups

On the 1st, 3rd, 5th and 7th day, after administering morphine to the rat for 1 h, the behavioural test demonstrated that 50% MWT of the rats showed no significant difference in the model saline and normal saline groups. On the 1st and 3rd day, after morphine injection, 50% MWT in the model morphine and normal morphine groups increased considerably, and significant difference was found compared with measurements before injection (both *P*<0.05), while comparisons among groups were not statistically different on the 1st and 3rd day (all *P*>0.05). On the 5th and 7th day, the 50% MWT in the model morphine and normal morphine groups decreased notably, and significant difference was found compared with those of the 1st and 3rd day (both *P*<0.05). Moreover, on the 5th day, the comparison between test results before injection and after injection was statistically different (both *P*<0.05), but on the 7th day, 50% MWT was back to the same level as before morphine injection, which was considered not statistically different (*P*>0.05). On the 1st, 3rd and 5th day, 50% MWT in the model morphine group was significantly higher than that in the model saline group (*P*<0.05) and 50% MWT in the normal morphine group was significantly higher than that in normal saline group (*P*<0.05). On the 7th day, there was no significant difference in 50% MWT between the normal morphine and normal saline groups, nor between the model morphine and model saline group (both *P*>0.05) (Figure 1). The above-mentioned results showed the successful establishment of BCP rat model with morphine tolerance.

### Comparisons of *miR-338* and CXCR4 mRNA and protein expression among four groups

**Figure 2:**
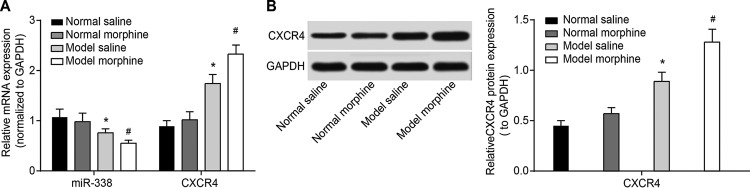
Comparisons of mRNA and protein expressions of *miR-338* and CXCR4 among four groups (**A**) Comparisons of mRNA expressionsof *miR-338* and CXCR4 detected by qRT-PCR among each group. (**B**) CXCR4 protein electrophoresis image and CXCR4 protein cartogram detected by Western blotting in each group; *P<0.05, compared with the normal saline group; ^#^*P*<0.05, compared with the model saline group.

In order to confirm the roles of *miR-338* and CXCR4 in BCP rat with morphine tolerance, ten rats taken from the normal saline, normal morphine, model saline and model morphine groups were killed after the successful establishment of BCP rat with morphine tolerance (on the 7th day after injection with morphine). L_3-4_ segments of the spines were removed to detect expression changes of *miR-338* and CXCR4. The result showed that CXCR4 mRNA expression in the model saline and model morphine groups was higher than those of the normal saline and normal morphine groups respectively (both *P*<0.05, Figure 2A). Also, *miR-338* mRNA expression in the model saline and model morphine groups was lower than those of the normal saline group and the normal morphine group respectively (both *P*<0.05, Figure 2A). According to the Western blotting (*P*<0.05, Figure 2B), this trend was consistent with the result of qRT-PCR (*P*<0.05).

### CXCR4 confirmed as a target gene of *miR-338*

**Figure 3:**
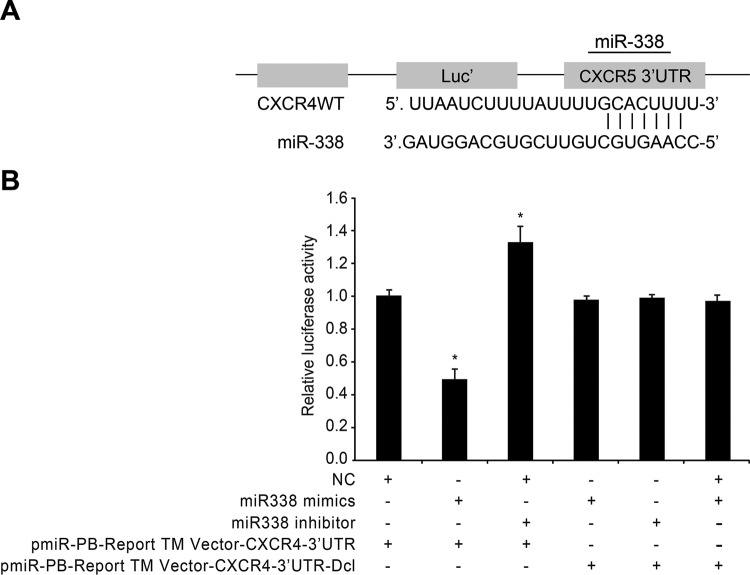
Comparisons of fluorescein activity in rat bone cancer cells after transfection of wild-type and mutant CXCR4-3′-UTR plasmids among four groups (**A**) The sequences for combined site of *miR-338* and CXCR4-3′-UTR region. (**B**) Luciferase assay results; *P<0.05, compared with other groups.

Biomedical database and TargetScan (target point analysing tool) were employed, and the gene structures were analysed by gene complementation theory, verifying that CXCR4 was one of the target genes of *miR-338* in bone cancer cells with the pre-experiment, and also that *miRNA-338* seems to play a biological role by identifying and combining with CXCR4 mRNA. In order to further confirm that CXCR4 is a direct target gene of *miR-338*, CXCR4-3′-UTR-WT, CXCR4-3′-UTR-Mut and *miR-338* mimics were co-transfected with miRNA-NC as an NC. The fluorescence value of each well was detected after 48 h. It was found that after *miR-338* mimics, 3′-UTR and mutant fluorescent enzyme reporter plasmid had co-transfected rat bone cancer cells, compared with the NC group, *miR-338* and wild-type plasmid showed decreased Renilla fluorescence enzyme activity, and as a result lower activity ratio of Renilla fluorescence enzyme to firefly luciferase activity (*P*<0.05). There was no significant difference in the effect of *miR-338* on mutant plasmid (*P*=0.404), as shown in Figure 3.

### Comparisons of MWT detected by behavioural test after lentivirus infection among four groups

**Figure 4:**
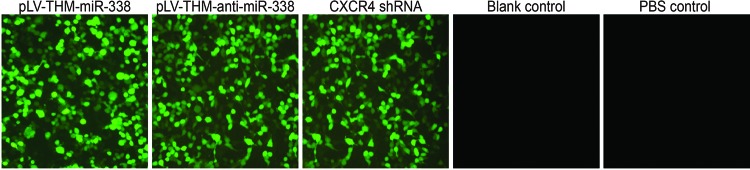
Virus infection level observed in rat spinal cord sections on the 14th day after virus injection among four groups

**Figure 5:**
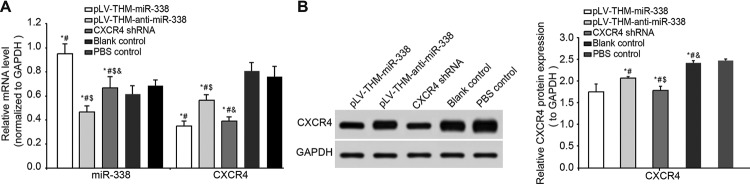
Comparisons of expressions of *miR-338* and CXCR4 after lentivirus infection (**A**) mRNA expressions of CXCR4 and *miR-338* in L_3-4_ spinal cord tissues detected by qRT-PCR in each group on the 14th day after virus suspension injection in BCP rats undergoing morphine tolerance. (**B**) Protein electrophoresis and protein changes of CXCR4 of L_3-4_ spinal cord tissues detected by Western blotting on the 14th day after virus suspension injection in BCP rats; *P<0.05, compared with the blank control group; ^#^*P*<0.05, compared with the PBS control group; ^∃^*P*<0.05, compared with the pLV-THM-*miR-338* group; ^&^*P*<0.05, compared with the pLV-THM-anti-*miR-338* group.

After the successful establishment of BCP rat model with morphine tolerance, the remaining 40 rats were randomly divided into pLV-THM-*miR-338*, pLV-THM-anti-*miR-338*, CXCR4 shRNA, blank control and PBS control groups, and intravenous injection of virus suspension of the same titre or equivalent sterile PBS solution was performed. On the 7th, 9th, 11th and 14th day after injection, 50% MWT was measured in each group and the results are shown in Table 2. There was no significant difference in 50% MWT between the blank control and PBS control groups. In the pLV-THM-*miR-338*, pLV-THM-anti-*miR-338* and CXCR4 shRNA groups, 50% MWT began to rise from the 9th day, reached its highest point on the 11th day and returned to the same condition as prior to lentivirus injection on the 14th day. On the 7th, 9th, 11th and 14th day after injection, 50% MWTs in the pLV-THM-*miR-338* and CXCR4 shRNA groups were significantly higher than those of the other four groups (all *P*<0.05), but an opposite result was observed in the pLV-THM-anti-*miR-338* group. There was no significant difference in 50% MWT between the blank control and PBS control groups (*P*>0.05). On the 14th day, after behavioural test, rats in each group were killed and L_5_ segment of the spines were removed, frozen and placed under fluorescent microscope to observe virus infection (Figure 4). When transfection efficacy reached an average of 80%, in the pLV-THM-*miR-338*, pLV-THM-*anti-miR-338* and CXCR4 shRNA groups, the titres of lentivirus were 4.8 × 10^8^ TU/ml, 5.5 × 10^8^ TU/ml and 6.9 × 10^6^ TU/ml respectively. The *miR-338* and CXCR4 mRNA expressions in L_3-4_ spinal tissues were detected with qRT-PCR on the 14th day (Figure 5A). CXCR4 protein expression change was measured with Western blotting and immunohistochemistry (Figure 5B), with results showing that *miR-338* expression of the pLV-THM-*miR-338* group was significantly higher than those of the CXCR4 shRNA, blank control and PBS control groups, while *miR-338* expression of the pLV-THM-anti-*miR-338* group was remarkably lower that those three groups (all *P*<0.05). There was no significant difference in *miR-338* expression among the CXCR4 shRNA, blank control and PBS control groups (all *P*>0.05). Expressions of CXCR4 mRNA and protein in the pLV-THM-*miR-338* group, pLV-THM-anti-*miR-338* and CXCR4 shRNA groups were lower than those of other groups (all *P*<0.05). There was no significant difference in CXCR4 mRNA and protein expressions between the blank control and PBS control groups (both *P*>0.05).

**Table 2:** The comparisons of 50% MWT of BCP rats with morphine tolerance on the 7th, 9th, 11th and 14th day after injection of virus suspension

Group	7th day	9th day	11th day	14th day
pLV-THM-*miR-338* group	4.14 ± 0.71*^†^	7.80 ± 0.82*^†^^‡^	8.84 ± 0.92*^†^^‡^^§^	4.77 ± 0.75*^†^^§^
pLV-THM-anti-*miR-338* group	3.85 ± 0.67*^†^	6.38 ± 0.79*^†^^‡^	8.01 ± 0.88*^†‡^^§^	4.09 ± 0.76*^†^^§^
CXCR4 shRNA group	4.03 ± 0.88*^†^	7.71 ± 0.96*^†^^‡^	8.69 ± 0.87*^†‡^^§^	4.74 ± 0.89*^†^^§^
Blank control group	5.48 ± 0.44	5.56 ± 0.48	5.69 ± 0.57	5.81 ± 0.48
PBS control group	5.52 ± 0.39	5.63 ± 0.54	5.74 ± 0.52	5.86 ± 0.43

Note: *P<0.05, compared with the blank control group; ^†^*P*<0.05, compared with the PBS control group; ^‡^*P*<0.05, compared with the 7th day; ^§^*P*<0.05, compared with the 9th day.

## Discussion

Cancer is one of the most serious threats to human survival, and 50–80% of cancer patients feel moderate or severe pain, especially those with advanced cancer [19]. Common tumours transfer easily to the bones, resulting in BCP. The patient’s body builds morphine tolerance after long-term morphine use, which reduces the analgesic effects [20]. Therefore, this present study aimed to explore the mechanism of *miR-338*’s regulation on CXCR4 in development of morphine tolerance in rats with BCP by establishing a BCP rat model with chronic morphine tolerance. It was found that *miR-338* and CXCR4 played important roles in morphine tolerance in BCP and that *miR-338* can inhibit CXCR4 to delay the formation of morphine tolerance in BCP.

The study found that after building morphine tolerance in BCP rat model, *miR-338* showed significantly lower expression, suggesting that *miR-338* may be related to the formation of BCP. miRNAs are active in cell proliferation, differentiation, apoptosis and metastasis, and they are often located at fragile sites and genomic regions of deletion and amplification observed in cancer. They can be oncogenic or tumour suppressive depending upon their downstream targets [21]. *miR-338* is located in chromosome 17q25 of *AATK* gene and plays vital roles in promoting cells apoptosis, neuron differentiation and neurite extension At G_0_/G_1_ stage, *miR-338* can inhibit cells’ proliferation, metastasis and invasion [22]. Since pain and morphine tolerance have similar signalling pathways, miRNA may also be involved in the development of morphine tolerance, and neurons in the dorsal horn of the spinal cord may also be key in pain conduction and development of morphine tolerance [23]. While *miR-338* is a kind of brain-specific miRNA [10] also expressed in spinal cord [24], it is safe to infer that *miR-338* is related to development of morphine tolerance. Researches show that *miR-338* expression was down-regulated in liver cancer and oral squamous cell carcinoma, and this reduced level of *miR-338* was closely related to malignant activities like cancer metastasis that can cause BCP [25–27]; thus we can infer that *miR-338* expression may also be down-regulated. According to Liang et al. [28], synthesized precursor miRNA can significantly reduce the expression of CXCR4, thus reducing the CXCR4/SDF-1 pathway-mediated tumour invasion and metastasis, suggesting that miRNA can be used as an upstream factor of CXCR4 pathway in the regulation of tumour metastasis. Furthermore, past studies have indicated that *miR-338* can affect cancer invasion and metastasis by reducing CXCR4 expression [29], so as to slow the development of BCP.

Our study also found that after generating morphine tolerance in BCP rat model, CXCR4 expression significantly increased, suggesting its importance in morphine tolerance development. CXCR4, also known as ‘fusin’, is a well-researched chemokine receptor [30]. Morphine use can lead to the increased expressions of the inflammatory factors, and pro-inflammatory factors have a tendency to increase pain sensitivity, namely, to suppress and significantly reduce the effect of acute opioid analgesic, and chemotactic factor CXCR4 has the same pro-inflammatory effect [15,31]. CXCR4 is mainly expressed in neuronal cell membrane of spinal dorsal horn, and its activation can generate downstream intracellular signalling pathways to neuron activation, a primary cause of hyperalgesia, indicating that CXCR4 is closely related to morphine tolerance [32]. Research displayed that CXCR4 signalling pathway blocking can reduce inflammation, reduce pain and delay the development of morphine tolerance. After morphine injection, CXCR4 expression in other studies increased, in line with the results of this research [15,33]. Furthermore, the present study confirmed that CXCR4 is a direct downstream target gene of *miR-338*, suggesting it plays a biological role by identifying and binding CXCR4 mRNA, providing a new insight into the development of morphine tolerance.

Based on a successful construction of BCP rat model, the present study of chronic morphine tolerance found that *miR-338* and CXCR4 played key roles in BCP morphine tolerance development. Furthermore, *miR-338* through targeted regulation of CXCR4 expression affected and delayed the development of morphine tolerance in BCP. Further studies are required, however, to fully understand the specific mechanisms of *miR-338* targeting of CXCR4.

## Abbreviations

AATK: apoptosis-associated tyrosine kinase
BCP: bone cancer pain
CVCR4: cxc chemokine receptor-4
CXCR4: CXC chemokine receptor-4
dh20: distilled water
DMEM: Dulbecco’s modified Eagle’s medium
L5: the fifth lumbar
MWT: mechanical withdrawal threshold
NC: negative control
qRT-PCR: quantitative real-time PCR
SD: Sprague–Dawley
SDF-1: stromal cell derived factor 1
SYBR: Synergy Brand
VSVO: Vector state-vector observation
WT: wild type
